# Educación permanente en salud para directivos como herramienta para combatir el racismo institucional en un municipio del noreste de Brasil

**DOI:** 10.18294/sc.2024.4905

**Published:** 2024-11-13

**Authors:** Matheus Guirra Martins Ferreira, Gilmar Mercês de Jesus, Etna Kaliane Pereira da Silva, Victor Bertino Silva, Mariana Costa da Silva

**Affiliations:** 1 Especialista en Salud Familiar. Programa de Residência Multiprofissional em Saúde da Família, Universidade Estadual de Feira de Santana. Feira de Santana, Bahia, Brasil. matheusfarmaufba@gmail.com Universidade Estadual de Feira de Santana Programa de Residência Multiprofissional em Saúde da Família Universidade Estadual de Feira de Santana Feira de Santana Bahia Brazil matheusfarmaufba@gmail.com; 2 Doctor en Educación Física. Docente, Universidade Estadual de Feira de Santana. Feira de Santana, Bahia, Brasil. gilmar.merces@uefs.br Universidade Estadual de Feira de Santana Universidade Estadual de Feira de Santana Feira de Santana Bahia Brazil gilmar.merces@uefs.br; 3 Doctora en Salud Pública. Docente, Centro das Ciências Biológicas e da Saúde, Universidade Federal do Oeste da Bahia. Barreiras, Bahia, Brasil. etnakaliane@gmail.com Universidade Federal do Oeste da Bahia Centro das Ciências Biológicas e da Saúde Universidade Federal do Oeste da Bahia Barreiras Bahia Brazil etnakaliane@gmail.com; 4 Especialista en Salud Familiar. Residente, Programa Multiprofissional Integrado em Saúde Hospitalar, Hospital Geral Roberto Santos. Salvador, Bahia, Brasil. bertinovictor@hotmail.com Hospital Geral Roberto Santos Programa Multiprofissional Integrado em Saúde Hospitalar Hospital Geral Roberto Santos Salvador Bahia Brazil bertinovictor@hotmail.com; 5 Magíster en Enfermería. Profesor, Universidade Estadual do Sudoeste da Bahia. Jequié, Bahia, Brasil. mariana.silva@uesb.edu.br Universidade Estadual do Sudoeste da Bahia Universidade Estadual do Sudoeste da Bahia Jequié Bahia Brazil mariana.silva@uesb.edu.br

**Keywords:** Educación Permanente, Racismo, Gestión en Salud, Brasil., Continuing Education, Racism, Health Management, Brazil

## Abstract

El artículo relata la experiencia de una acción de educación permanente en salud sobre racismo institucional con profesionales de la salud del Sistema Único de Salud (SUS), que ocupan cargos de gestión en un municipio del interior de Bahía, región nordeste de Brasil. Se realizó un taller en septiembre de 2022, basado en una metodología problematizadora denominada Arco de Maguerez, llevada a cabo por residentes del Programa Multiprofesional en Salud Familiar. El taller contó con la participación de diez gestores, siendo la mayoría mujeres. Los gestores, a pesar de reconocer la presencia y el impacto del racismo en los servicios de salud, encuentran dificultades para visibilizar y combatir el racismo en su dimensión institucional. Fue notable el discurso recurrente alineado con el mito de la democracia racial, y se observó un mayor compromiso de las mujeres negras a lo largo del taller. La educación permanente en salud se muestra como una importante estrategia de combate al racismo, necesaria para ampliar los debates de este tipo dentro de los servicios de salud.

## Abstract (en)

This article discusses the experience of a permanent health education initiative focused on institutional racism with health professionals from the Brazilian Unified Health System (SUS) holding management positions in a municipality in the interior of Bahia, in Northeastern Brazil. A workshop was held in September 2022, based on a problematizing methodology called the Maguerez Arc, conducted by residents of the Multiprofessional Family Health Program. Ten managers participated in the workshop, most of whom were women. Despite acknowledging the presence and impact of racism in health services, managers find it challenging to recognize and address racism in its institutional dimension. A recurring discourse aligned with the myth of racial democracy was notable, and a higher level of commitment among Black women was observed throughout the workshop. Permanent health education emerges as an essential strategy to combat racism, necessary to expand such discussions within health services.

## INTRODUCCIÓN

En Brasil, el racismo es un factor estructurante de inequidades en salud^1^^,^^2^, pero a pesar de la significativa contribución de los movimientos negros en la reforma sanitaria, el tema fue poco internalizado en el Sistema Único de Salud (SUS) de forma práctica^2^. En este contexto, fue un gran logro la Política Nacional de Salud Integral de la Población Negra (PNSIPN), publicada en 2009 e incorporada como ley en el Estatuto de la Igualdad Racial en 2010. En esta política, el Estado brasileño reconoce la presencia y el impacto del racismo institucional en el SUS, estableciendo directrices y estrategias para enfrentar el racismo y reducir las desigualdades raciales en el área de la salud. Al tratarse de una política transversal, la PNSIPN abarca la formación de los profesionales de la salud, la gestión participativa, el control social y la garantía de acciones de promoción, prevención y protección a la salud^3^^,^^4^.

El proceso salud-enfermedad está relacionado con las condiciones sociales, económicas, culturales, ambientales y políticas en las cuales los individuos se encuentran^5^^,^^6^. Diversos estudios señalan que los individuos negros presentan, en comparación con la población blanca, peores autopercepciones de salud, mayor exposición a condiciones de vida precarias, menor acceso a los servicios de salud y un mayor número de muertes por causas evitables^7^^,^^8^. Estas desigualdades se hicieron aún más evidentes durante la pandemia del SARS-COV-2, en la cual la población negra y periférica estuvo más expuesta al virus y sus consecuencias que la población blanca^9^^,^^10^^,^^11^. En este sentido, en 2011, la Organización Mundial de la Salud (OMS) propuso un modelo que considera la distribución de renta, la raza/etnia y el género como determinantes estructurales, reconociendo así el impacto de estos determinantes sociales en el contexto de salud de la población^5^.

De acuerdo con Jones^1^, el racismo no puede reducirse a una patología individual, moral o psiquiátrica. Es un sistema que, basándose en el fenotipo, genera una serie de desventajas para algunos individuos y grupos, mientras proporciona ventajas para otros. Por lo tanto, el racismo se manifiesta en tres dimensiones: individual, institucional y estructural^12^. Al discutir la dimensión institucional del racismo, nos referimos a las disparidades en el acceso a servicios y oportunidades que resultan de la estructura de las instituciones, incluidas sus políticas, protocolos, normas y prácticas^1^. Este tipo de racismo a menudo es ignorado, ya que no es perpetrado por un individuo específico, sino que está enraizado en la propia legislación^1^^,^^2^. En consecuencia, el racismo puede resultar en diferentes niveles de desigualdad en la atención de salud, reflejándose en el acceso a los servicios de salud, en la calidad de la atención brindada a estos grupos y, de forma más amplia, en las condiciones de vida a las cuales estos individuos están expuestos^1^^,^^13^.

Así, es extremadamente importante la creación de espacios transformadores que eliminen o reduzcan los impactos del racismo en la atención a la salud. La educación permanente en salud (EPS) representa una de las prácticas que posibilita estos espacios, ya que tiene como objetivo promover la reflexión crítica y la (re)construcción de conocimientos a partir del proceso de trabajo^14^. Brasil presenta una estructura institucionalizada de formación y desarrollo de los trabajadores del SUS basada en la educación permanente en salud, adoptando el aprendizaje en el trabajo, donde aprender y enseñar se incorporan al cotidiano de las organizaciones y al trabajo^15^.

La educación permanente en salud se basa en el aprendizaje significativo, partiendo de la realidad concreta de los trabajadores, interconectando el entendimiento que ellos poseen acerca del proceso de trabajo, sus valores y los malestares generados a partir de los desafíos vividos para transformar los contextos y procesos de trabajo de forma participativa. La educación permanente en salud, por lo tanto, se propone construir entre los profesionales un proceso de autoanálisis y de gestión participativa, generando cambios en el escenario de acción y, principalmente, haciendo que el colectivo sea capaz de construir y reconstruir constantemente los escenarios de forma autónoma y continua a partir de lo que se aprende y experimenta dentro del servicio, considerando las necesidades de salud de las personas y poblaciones^15^^,^^16^.

Ante esto, el presente artículo tiene como objetivo relatar la experiencia de una acción de educación permanente en salud sobre racismo institucional con profesionales de salud del Sistema Único de Salud (SUS) que ocupan cargos de gestión en un municipio del interior de Bahía, región nordeste de Brasil.

## MÉTODOS

Se trata de un relato de experiencia, construido a partir de la vivencia en el proceso de construcción y aplicación de un taller titulado “Combate al Racismo Institucional en el Sistema Único de Salud”. El relato de experiencia puede entenderse como un tipo de estudio que busca describir vivencias y/o intervenciones y, a partir de la fundamentación teórica, llevar a una reflexión crítica^17^.

El taller fue llevado a cabo por los residentes del programa de Residencia Multiprofesional en Salud Familiar de la Universidade Estadual de Feira de Santana (RMSF-UEFS) y tuvo como público objetivo a los profesionales de la salud que asumen cargos de gestión en el SUS, en un municipio del interior de Bahía. El programa de residencia cuenta con diez plazas para profesionales de cinco categorías, que son: farmacia, psicología, educación física, enfermería y odontología. En el primer año, se asignan los residentes a servicios de atención primaria de salud del municipio. En el segundo año, el enfoque se centra en acciones de gestión, que abarcan sectores relacionados con la gestión municipal y regional del SUS. El taller fue organizado en el segundo año de la residencia, a partir de que las y los residentes identificaran una ausencia de acciones vinculadas a la Política Nacional de Salud Integral de la Población Negra en el municipio.

El municipio bahiano, en el cual se realizó el taller, pertenece a la macrorregión de salud Centro-Leste. De acuerdo con el Instituto Brasileño de Geografía y Estadística, tenía en 2022 una población estimada de más de 50 mil habitantes, de la cual el 87% de la se declaraba como negra (parda o *preta*).

El taller se llevó a cabo en el mes de septiembre de 2022. Los profesionales que ocupaban cargos de gestión en los servicios de salud del SUS fueron invitados al taller mediante una invitación impresa entregada de manera individual. Además, se enviaron recordatorios sobre el encuentro a través de grupos en una aplicación de mensajería instantánea. El taller se realizó en el turno matutino, en el auditorio de la Secretaría Municipal de Salud.

Sobre la base de reconocer la importancia de la construcción de conocimientos que surjan del propio proceso de trabajo y en las problemáticas vividas por los participantes, el taller se estructuró a partir del Arco de Maguerez. Dicha metodología tiene como principal objetivo construir el conocimiento de forma activa y mediante problematizaciones y conexiones con la realidad vivida por los individuos. Así, el Arco de Maguerez comienza con la observación de la realidad, con el fin de identificar características y enumerar posibles problemas. Luego, se procede a una reflexión sobre los factores involucrados en esa situación, que se utilizan como puntos claves para avanzar hacia la teorización, en la que se construye un análisis más profundo y elaborado de la situación analizada. Finalmente, se busca plantear hipótesis de solución para la situación y aplicarlas a la realidad^18^.

**Figura 1:**
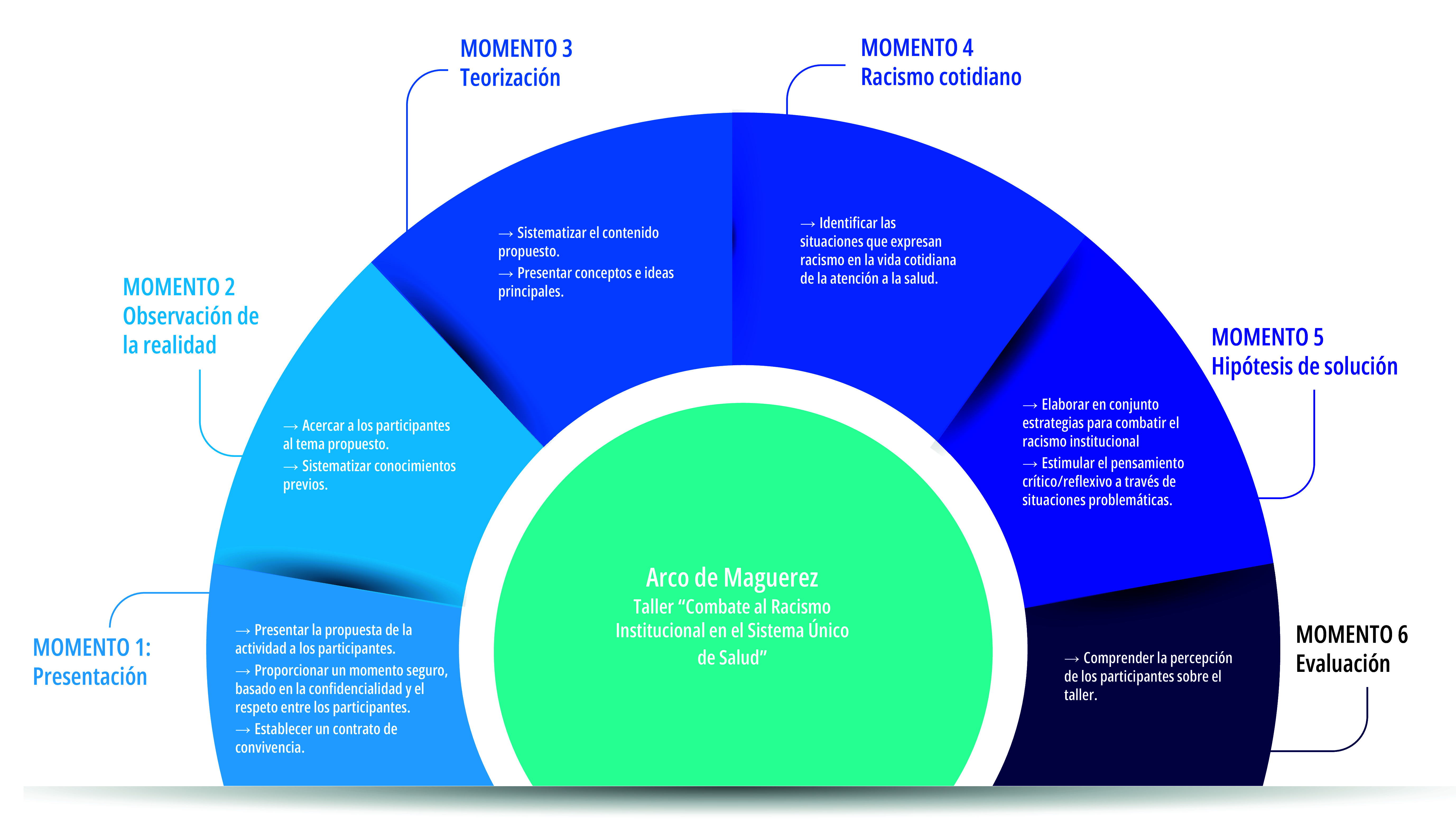
Arco de Maguerez del taller “Combate al Racismo Institucional en el Sistema Único de Salud” y objetivos de cada momento propuesto. Programa de Residencia Multiprofesional en Salud Familiar de la Universidade Estadual de Feira de Santana (RMSF-UEFS), Bahia, Brasil, 2022.

A partir de esto, el taller se dividió en momentos (Figura 1): el momento 1 incluyó la presentación de la propuesta y los objetivos del taller, así como la creación de un contrato de convivencia que garantizara un ambiente basado en el respeto y el secreto, creando un entorno más propicio para la expresión de ideas y pensamientos sobre el tema. 

El momento 2 tuvo como objetivo acercar a los participantes al tema del taller y sistematizar los conocimientos previos del grupo. Para ello, se plantearon preguntas orientadoras como “¿Qué es raza?”, “¿qué es racismo?”, “¿cómo se manifiesta el racismo?” y “¿cómo impacta el racismo en el cuidado a los usuarios?”. A medida que se presentaban las preguntas, el grupo expresaba sus ideas y las sistematizaba en los carteles correspondientes a cada pregunta.

En el momento 3 se realizó un bloque expositivo, en el cual se abordaron temas como: antecedentes históricos, dimensiones del racismo, políticas de salud dirigidas a la población negra, registro del ítem raza/color, condiciones de salud más prevalentes en la población negra y el acogimiento dentro de los servicios de salud. 

En el momento 4, se invitó a los participantes a reflexionar sobre los servicios locales de salud y a identificar posibles situaciones/problemas que expresaran racismo en ese contexto; posteriormente, se enumeraron las causas de cada uno de estos problemas.

A continuación, en el momento 5, los participantes se dividieron en dos grupos y recibieron casos ficticios con situaciones problemáticas. El objetivo fue que los grupos discutieran posibles estrategias para abordar la situación en cuestión, permitiendo la contextualización del tema tratado a lo largo del taller y fomentando un pensamiento crítico-reflexivo en situaciones que podrían ocurrir dentro del contexto de atención a la salud. Finalmente, en el momento 6, se invitó a los participantes a expresar su evaluación del taller.

Para el registro de la actividad, a partir de la propuesta y los objetivos de cada momento, se construyó un cuaderno de campo que guió el registro de las percepciones y las observaciones de los residentes. Las dinámicas contaron con carteles que permitieron la sistematización de las ideas de forma visual, para que todo el grupo pudiera verlas. 

Al tratarse de un estudio teórico, de una situación que surgió de manera espontánea en la práctica profesional de los residentes, y con el propósito de sistematizar sus experiencias en la acción educativa sin el presente relato no requiere aprobación por parte de un Comité de Ética en Investigación.

La inclusión del programa de residencia en los servicios del municipio implica, entre otras actividades, la realización de actividades de formación con los profesionales allí destinados. Así, este fue uno más de los talleres aprobados por la institución que se llevaron a cabo a lo largo del curso con diferentes temáticas. Luego de su realización, se identificó la importancia y profundidad del momento formativo y se decidió escribir un artículo dando cuenta de esta experiencia, preservando la identidad de las personas participantes. El trabajo contó con la aprobación tanto del programa de residencia en el que parte de este trabajo se presentó como requisito para completar la residencia por parte de uno de los autores, así como de la institución en la que se desarrolló la actividad.

## RESULTADOS

El taller contó con la participación de diez profesionales de la salud que ocupaban cargos de gestión en los servicios de salud del SUS: secretaria municipal de salud, coordinadora de atención básica, coordinadora general de vigilancia en salud, representante de vigilancia sanitaria, representante de vigilancia epidemiológica, coordinador de asistencia farmacéutica, directora administrativa del hospital municipal, coordinadora de farmacia del hospital municipal, coordinadora del equipo multiprofesional de apoyo matricial del municipio y representante del sector de planificación en salud. Cabe resaltar que el número de participantes varió a lo largo del taller; cinco profesionales se ausentaron en distintos momentos (un hombre blanco, tres mujeres blancas y una mujer negra) y cinco participaron en todos los momentos (cuatro mujeres negras y una mujer blanca).

En el momento 1, a pesar de ser una instancia introductoria cuyo objetivo era presentar la propuesta del taller y establecer un contrato de convivencia, algunos participantes compartieron relatos sobre sus percepciones acerca del racismo dentro de los servicios de salud. Una participante señaló que había presenciado diversas situaciones de racismo, pero que este no se asociaba únicamente al color de piel, sino también a las condiciones de vida. Sin embargo, otra participante contrastó con relatos sobre cómo el racismo se manifestaba en la sociedad y se reproducía también en las instituciones, mencionando que la abolición ocurrió solo en la ley y que las personas negras en Brasil aún vivían en un proceso violento de esclavización, además de destacar la importancia de contar con espacios para debatir el tema.

En el momento 2, a partir de las discusiones y las palabras escritas en los carteles, fue posible observar que, en cuanto al concepto de raza, la mayoría entendió que se refería a un grupo de personas con características comunes. A partir de esto, una participante señaló que todos los individuos son iguales, lo cual generó disenso en el grupo, ya que algunos participantes defendieron la idea de que no todos los individuos son iguales.

En cuanto a las definiciones de racismo, surgieron términos como “discriminación”, “explotación”, “exclusión”, “falta de respeto”, “prejuicio” y “violencia”. Motivados por los residentes, los participantes fueron cuestionados sobre si creían que había racismo en Brasil, y el grupo estuvo de acuerdo en que sí. A partir de esto, se invitó al grupo a reflexionar sobre cómo se manifestaba ese racismo, obteniéndose respuestas como: “A veces sutil”, “ofensiva”, “violenta”, “juicio”, “explotadora”, “exclusión”, “falta de empatía”, “micro y macro violencias”.

A continuación, se les preguntó también sobre la existencia de racismo dentro de los contextos de los servicios de salud, y el grupo coincidió en que existía racismo. Una participante expresó que “al ser estructural, el racismo está en todos los espacios, incluidos los servicios de salud”. Cuando se les cuestionó cómo ese racismo impactaba en el cuidado de los usuarios, respondieron: “diferencias en el acogimiento/tratamiento”, “negligencias”, “exclusión” y “desistencia por parte de los usuarios”. Estas preguntas llevaron a algunos participantes a relatar situaciones de racismo que habían presenciado en los servicios de salud, e incluso una participante señaló que, en una situación, el hecho de ser negra y haber vivido experiencias similares fue crucial para ponerse en el lugar del usuario y resolver de la mejor manera la demanda.

El momento 3, expositivo/dialogado, abordó el contexto histórico de la construcción de la raza y el racismo en Brasil, las dimensiones del racismo, la relación entre raza y salud, y la política nacional de salud integral de la población negra. A partir de esto, el grupo discutió cómo el racismo también afecta a los profesionales de la salud, impactando desde su relación con los usuarios -quienes, en ocasiones, no los reconocen como profesionales de nivel superior y/o no aceptan ser atendidos por ellos- hasta su relación con sus propios colegas, dado que los profesionales negros también se veían afectados por el racismo dentro de los servicios.

En las intervenciones del grupo, también surgió una discusión sobre el proceso de construcción de identidad y raza. Una profesional expresó su percepción de que en Brasil no existían negros, ya que “todos tienen la sangre mezclada”, a lo que otra participante añadió que “en Brasil todos tienen un pie en la *senzala*” [barrio de esclavos]. Además, el grupo destacó la importancia de abordar este tema en las escuelas y en la educación familiar. A pesar de que uno de los objetivos del tercer momento era dialogar sobre la política nacional de salud integral de la población negra, reiteradamente surgieron comentarios que tendían a suavizar el racismo en Brasil, como si las razas convivieran en armonía. Al mismo tiempo, algunos participantes enfatizaban el racismo estructural, aligerando la responsabilidad individual. Los residentes se sintieron frustrados debido a que el diálogo no profundizó en lo que respecta a la política de salud.

El momento 4 tenía como propuesta principal que el grupo identificara situaciones en las que el racismo se manifestara dentro del contexto de los servicios locales de salud. Entre las situaciones enumeradas, cabe destacar el no reconocimiento de las mujeres negras como profesionales y/o líderes dentro de un servicio de salud, siendo esta una cuestión planteada por mujeres negras y reforzada con relatos de experiencias personales. Sin embargo, fue evidente la dificultad del grupo para visualizar qué situaciones expresaban racismo específicamente dentro de sus vivencias profesionales, llevando el debate a una dimensión más global, con respuestas como: “ser mujer, pobre y líder”, “tener el cabello duro” y “clase social”. Al preguntarles sobre las causas de estas situaciones racistas, señalaron la falta de empatía y de educación familiar, la falta de conocimiento y la falta de atención a la necesidad de cambio. En este momento, una participante blanca comentó que “el racismo no ocurre solo con los negros”, relatando experiencias de discriminación por ser residente de otro municipio, cuestiones de clase como no ser llamada doctora y no tener un piso salarial establecido. Es importante destacar que dicha participante en varias ocasiones centralizó el debate e incluso invalidó en algunos momentos los relatos aportados por participantes negras.

En el momento 5, se formaron dos subgrupos y cada uno recibió una situación problemática para debatir y presentar a los restantes integrantes del taller. En el caso 1, se narró una situación en la que una enfermera, recién llegada al servicio, relató que una usuaria se resistía a acudir al servicio, debido a que había vivido una situación de racismo, con otra profesional del servicio, por su vestimenta asociada a una religión de matriz africana. En el caso 2, se describió una situación que involucraba casos de racismo en el proceso de declaración de raza/color durante la anamnesis, así como la ausencia de esta información en gran parte de los registros de usuarios de un servicio.

A pesar de que los casos fomentaron una problematización inicial sobre las situaciones presentadas, ambos grupos propusieron únicamente acciones de educación en salud como estrategia de enfrentamiento. Por lo tanto, fue necesario que los residentes provocaran algunos debates sobre la intolerancia religiosa y la importancia de completar el ítem de raza/color para la implementación de políticas públicas eficaces. A partir de esto, el grupo debatió sobre las dificultades de los usuarios para declarar su raza/color. A pesar de que las situaciones problemáticas eran complejas y permitían una diversidad de intervenciones y discusiones, al tratarse de un momento avanzado del taller, se notó que los participantes ya estaban inquietos y con prisa por finalizar.

Al finalizar, en el momento 6, se incentivó al grupo a realizar una evaluación verbal del encuentro, destacándose la importancia del espacio formativo y la necesidad de ampliar este debate a otros sectores. El ambiente creado a lo largo del taller favoreció la expresión de opiniones y experiencias, surgiendo en varios momentos relatos de profesionales que habían experimentado de forma directa o indirecta los impactos del racismo en su quehacer profesional. Sin embargo, hubo una gran rotación de participantes y, en ciertos momentos, se observaron conversaciones paralelas a la discusión propuesta en el taller.

## DISCUSIÓN

Este relato presenta los resultados de la implementación de una acción de educación permanente en salud, dirigida a profesionales en cargos de gestión en el ámbito de la salud, como estrategia para enfrentar el racismo institucional. En general, los resultados revelaron una baja adhesión de los profesionales a la propuesta, especialmente aquellos identificados como blancos. Del total de profesionales que integraron el taller, solo cinco profesionales participaron activamente en todas las acciones desarrolladas, siendo cuatro de ellos mujeres negras. No obstante, entre los profesionales comprometidos con la actividad, surgieron debates importantes. El grupo reconoció el racismo institucional y estructural, aunque mostró dificultad para visualizar cómo ese racismo se manifiesta en su contexto profesional. Cabe destacar también el reconocimiento del profesional de salud negro como víctima del racismo, ya sea por parte de colegas o usuarios, y la existencia de intersecciones con otras opresiones. Sin embargo, el grupo también expresó discursos marcados por el no reconocimiento de razas distintas en Brasil.

Según nuestro conocimiento, pocos trabajos se proponen discutir o relatar experiencias sobre la formación en racismo en el ámbito de la salud en la bibliografía científica. Así, a través de la educación permanente en salud, esta experiencia dio los primeros pasos en línea con lo establecido por la Política Nacional de Salud Integral de la Población Negra (PNSIPN)^3^, de manera de incorporar el tema en los momentos de formación de los profesionales del municipio y propiciar reflexiones sobre la necesidad de implementar acciones en consonancia con la política.

A pesar de que la PNSIPN fue instituida en 2009, aún existen diversas barreras para la adhesión e implementación de la política en los municipios. Entre las principales dificultades cabe destacar el desconocimiento de los profesionales sobre la política, la falta de reconocimiento de la importancia del tema y de los impactos del racismo en el proceso de atención, así como la ausencia de estrategias para monitorear y evaluar acciones e indicadores de salud desagregados por raza/color^4^^,^^19^. En Bahía, la PNSIPN fue instituida mediante el Decreto 14720, del 29 de agosto de 2013; sin embargo, diversos estudios evidencian las dificultades para asegurar la capilaridad de las acciones hacia los municipios del interior, limitándose muchas de ellas a la capital, y no existen documentos oficiales para monitorear y evaluar la política en el Estado^20^^,^^21^.

En este sentido, el fortalecimiento del SUS implica el desarrollo de acciones que preparen a los profesionales para una actuación que garantice los principios del SUS, incluso para la población negra^19^. La educación permanente en salud, al ser una herramienta que facilita el aprendizaje a través del diálogo y la reflexión dentro del propio proceso de trabajo, es una aliada importante en el proceso de formación profesional y en la transformación de sus prácticas^14^^,^^22^.

A lo largo del taller, aunque los profesionales reconocieron la existencia del racismo dentro de las instituciones, los relatos y discusiones no superaron la perspectiva del racismo como un sesgo individual. Esto se debe, principalmente, a que el racismo opera de forma que naturaliza los impactos y desventajas generadas a la población negra^2^ y, desde la perspectiva del racismo institucional, esto se torna aún más sutil, ya que no se deriva de actos declarados y/o asociados a la acción de un individuo, operando de forma difusa en el cotidiano de los servicios^1^^,^^2^^,^^12^.

Almeida^12^ propone que el racismo puede estructurarse en tres dimensiones: individual, institucional y estructural. La dimensión individual parte del entendimiento de que el racismo se atribuye a individuos o grupos específicos, y está más relacionada con la discriminación explícita y directa. La concepción institucional y estructural, en cambio, considera el racismo no como una manifestación anormal y/o patológica, sino como un eje central y estructurante de la organización social^12^. A pesar de las dificultades para percibir el racismo en su forma institucional, sus impactos terminan siendo evidentes a partir de las inequidades generadas^23^. Por ello, Jones^1^ subraya la importancia de medir el racismo institucional mediante la documentación de las diferencias en el acceso a bienes y servicios, y mapear los factores que estructuran y perpetúan esta lógica. En esta línea, Cooper *et al*.^24^ proponen un diagrama que ofrece posibles caminos de intervención para hacer que la atención en salud sea más equitativa para los grupos raciales y étnicos. Según los autores, es necesario considerar las condiciones personales/familiares, financieras y estructurales que pueden alejar a determinados grupos de los servicios de salud, así como la capacidad de los profesionales para brindar una atención resolutiva y contextualizada con las condiciones culturales de esos individuos^2^^,^^24^.

Durante el taller, se observó otro aspecto del racismo dentro de los servicios de salud: la discriminación de los propios profesionales, perpetrada tanto por sus pares como por usuarios del servicio de salud. Entre los relatos de los participantes, cabe destacar la insistencia en experiencias en las cuales las personas usuarias no reconocían a las y los profesionales negros como personal con formación de nivel superior e, incluso, en algunas ocasiones mostraban rechazo a recibir atención por parte de profesionales negros. A pesar de que la PNSIPN reconoce el sesgo interpersonal del racismo dentro del propio equipo de salud^3^, en la bibliografía hay una escasez de estudios que se enfoquen en el impacto de este aspecto específico del racismo en el proceso de trabajo de los equipos y en la salud mental de los profesionales negros.

Estos relatos también demostraron que, además del aspecto racial, las violencias hacia las profesionales también incluían aspectos relacionados con el género; por ejemplo, se mencionó la falta de aceptación de algunos profesionales para ser liderados por mujeres negras. En este contexto, es importante tener en cuenta que las mujeres constituyen una parte significativa del cuerpo de profesionales de la salud que trabajan en el SUS^2^, como se observó en el servicio de salud en el que se realizó el taller, donde las mujeres representaron el 80% de las personas participantes. Las mujeres negras, en particular, están más expuestas a los impactos del racismo estructural, observándose diferencias significativas en los indicadores de salud en comparación con las mujeres blancas^13^^,^^25^.

Es sumamente importante, por lo tanto, que el debate sobre el racismo en salud se realice desde la perspectiva de la interseccionalidad. Según Akotirene^26^, este término se relaciona con la superposición de diferentes estructuras identitarias, que culminan en la colisión de distintos tipos de opresión. Así, las mujeres negras enfrentan barreras no solo por el color de su piel, sino también por el género, además de otras posibles opresiones. A pesar de la relevancia del debate interseccional en el ámbito de la salud, una revisión sistemática realizada por Oliveira y Kubiak^13^ señaló que existe una escasez de materiales que aborden la salud de la población negra con un enfoque de género. Y aunque son escasos, los estudios analizados por los autores muestran que las mujeres negras presentan los peores indicadores en salud.

Otro punto destacable fue la repetición de discursos que afirmaban que “todos son iguales” y la negación de la existencia de razas en Brasil. Este tipo de discurso fue difundido en la sociedad brasileña a partir de las políticas de blanqueamiento y del mito de la democracia racial^27^, que postulaba la mezcla racial como un rasgo fundamental de Brasil, que se percibía como un país en el que las razas convivían en armonía total. Esta idea termina por silenciar la exclusión social vivida por la población negra e indígena del país y refuerza el proceso de asimilación y/o aculturación de las identidades y vivencias de estas poblaciones, además de despojar a estos grupos de sus subjetividades^28^^,^^29^.

La negación de las distinciones raciales en Brasil ocurre de manera estratégica, casi siempre como respuesta a las políticas afirmativas que buscan reparar los impactos del racismo. La interiorización de esta idea en la sociedad brasileña contribuye a sostener y otorgar estabilidad a un sistema perverso^27^. Así, estos discursos acaban construyendo un racismo “cordial”, en el que la raza no parece ser un factor estructurante y las tensiones raciales y sus consecuencias quedan protegidas por un pacto de silencio que utiliza el mestizaje como principal argumento^30^^,^^31^. El mestizaje es una característica importante de la sociedad brasileña y, aunque no ha eliminado las tensiones raciales que atraviesan las estructuras sociales, ha hecho que la forma en que opera el racismo sea más compleja^29^^,^^31^. Así, a diferencia de la lógica birracial observada en otros países, el racismo en Brasil opera reconociendo gradaciones en la presencia/ausencia de características negras^27^^,^^30^.

Además, la falta de reconocimiento de las diferencias raciales impuestas por la construcción social y la falta de una atención diferenciada que garantice el acceso de esta población a sus derechos van en contra de lo establecido en la PNSIPN y violan el principio doctrinario de la equidad en el SUS. La equidad se entiende como un principio que busca una distribución desigual para los desiguales, reconociendo que los sujetos y grupos parten de experiencias diferentes y, por tanto, tienen demandas distintas^32^^,^^33^. En este sentido, la PNSIPN es un marco importante en la implementación de estrategias que buscan garantizar la aplicación de la equidad en la atención a la población negra, posibilitando la reducción de las inequidades que afectan directamente los indicadores de salud de este grupo^3^. Por tanto, es fundamental que los profesionales del SUS sean capaces de reconocer las diferencias y, más aún, de garantizar una atención que tenga en cuenta las diferencias en el proceso de cuidado, fortaleciendo un sistema no solo universal, sino también equitativo.

A lo largo de la actividad, se observó un mayor compromiso de las personas participantes identificadas como mujeres negras, incluyéndose una serie de relatos personales de cómo el racismo atraviesa sus experiencias personales y profesionales. En la misma medida, ciertos comportamientos sugirieron un posible malestar entre las personas blancas, como dispersión en el tema y abandono del taller por parte de este grupo. Según Grada Kilomba^34^, existe una incomodidad en el grupo que históricamente ha ocupado una posición de superioridad al enfrentarse con narrativas propuestas por personas negras, especialmente porque revelan temas que fueron fuertemente reprimidos para mantenerlos en una posición de confort y privilegio. También existe un desconocimiento de la blancura como un ser racializado, percibiéndose ser blanco como algo automático y natural^35^^,^^36^. Esta idea es reforzada por discursos que ignoran que las personas blancas también tienen un papel en los conflictos raciales, incluso en lo que respecta a las herencias del período esclavista^36^^,^^37^^,^^38^. Así, muchas veces, los individuos blancos se perciben ajenos a las desigualdades raciales, sus consecuencias y se desresponsabilizan de la lucha contra el racismo^36^^,^^38^.

De las cinco participantes que permanecieron hasta el final del taller, solo una de ellas se definió como mujer blanca. A pesar de que esta participante expresó reconocer la importancia de la discusión sobre el tema y la necesidad de combatir el racismo, incluso mencionando a diversas pensadoras negras, terminó dominando el debate y, en algunos momentos, deslegitimando los relatos aportados por las demás. En otras ocasiones, relativizó el racismo a través de otras discriminaciones relacionadas con la clase social, la categoría profesional y el lugar de origen. Aunque su discurso parecía asumir una postura antirracista, en realidad reafirma el pacto narcisista de la blancura que, según Maria Aparecida Bento^37^^,^^38^, es un acuerdo de silencio entre las blancuras, fundamental para mantener el sistema. Así, aunque se reconozca la existencia y el impacto del racismo, persiste una protección sobre los privilegios que esta estructura otorga a ciertos grupos.

Entre las limitaciones del proceso de implementación de la acción, cabe destacar el corto período de tiempo del taller para abordar un tema complejo y aún poco discutido en los momentos de formación de estos profesionales. Este factor se debe principalmente a la imposibilidad de que los profesionales dispongan de más de un turno semanal para participar en la actividad sin afectar el proceso de trabajo. Otra limitación fue el lugar en el que se realizó el taller; ya que, al ser el lugar de trabajo de los profesionales, se produjeron varias interrupciones debido a demandas externas a la actividad. Además, la ausencia de participantes varones negros en el taller generó una brecha en cuanto a su percepción del tema y la falta de relatos sobre cómo el racismo atraviesa su práctica profesional.

A pesar de estas limitaciones, el taller fue un paso importante para la reflexión de los profesionales sobre el reconocimiento del racismo, sus impactos y la necesidad de implementar la PNSIPN. Es relevante destacar que la inclusión de la formación sobre este tema en los servicios está contemplada en la PNSIPN, por lo que el taller constituye una forma de implementar acciones en consonancia con la propia política.

## CONSIDERACIONES FINALES

El taller de combate al racismo institucional con profesionales en cargos de gestión en el sistema único de salud fue una estrategia poderosa para fomentar el pensamiento crítico sobre el tema, así como para delinear posibles estrategias para combatir el racismo dentro de los servicios de salud. La educación permanente en salud, al problematizar el proceso de trabajo a partir de las experiencias de los profesionales, se muestra como una herramienta importante para ampliar la mirada y el quehacer profesional crítico-reflexivo. No obstante, el debate aún necesita ampliarse, tanto en términos de generar otros momentos y acciones sobre el tema con estos profesionales, como en replicar el taller para un grupo más amplio de profesionales.

El Arco de Maguerez demostró ser una herramienta metodológica valiosa para construir y sistematizar conocimientos a partir de la problematización de la realidad de los participantes, en consonancia con los objetivos de la educación permanente en salud.

Es importante destacar que este relato es un recurso significativo para el análisis y la planificación de acciones futuras, con el fin de abordar de manera más profunda aquellas cuestiones que aún requieren una mayor asimilación por parte de los profesionales, especialmente en lo que respecta a la implementación real de acciones alineadas con la Política Nacional de Salud Integral de la Población Negra.
